# Gastric dilatation in women with eating disorder. Diagnosed and control by ultrasound

**DOI:** 10.1186/2036-7902-7-S1-A34

**Published:** 2015-03-09

**Authors:** Jose Retamal, Ramon Nogue, Juan Fabregat, Laura Clavel, Rosa Vilella

**Affiliations:** Emergency Department, Hospital Montserrat, Lleida, Spain

**Keywords:** Emergency Department, Constipation, Eating Disorder, Nasogastric Tube, Gastric Content

## Gastric dilatation in women with eating disorder. Diagnosed and control by ultrasound

Gastric dilatation may be an important complication in patients with eating disorders, particularly bulimia, which requires early diagnosis. We present a case in which it could be diagnosed using utrasonography in emergency department.

## Anamnesis

19 year old woman consulted our emergency department for abdominal pain within 3 hours of onset, without nausea, diarrhea or constipation, having presented a unique bilious vomit. He denies any personal history of interest.

## Physical examination

FC: 88 bpm, BP: 113/75 mmHg, SO2: 98%. Abdominal distention with diffuse signs of peritonism.

## Investigations

The blood analysis showed no remarkable changes.

The abdomen radiography showed a diffuse absence of air throughout all the abdomen. (image)

## Evolution

Assessed by the surgeon, who after considering the episode as an acute abdomen, indicated the need for surgical intervention. It was decided to perform a bedside ultrasound previous to the surgery, detecting an intraluminal structure without peristalsis, extending from the epigastrium to hypogastrium, suggestive of gastric dilatation. Study was completed by abdominal CT scan which confirmed the diagnosis.

Following the new findings anamnesis was performed again. The patient told a high intake of yogurt and large amounts of water in the hours before the onset of symptoms. She explained previous episodes that resolved after vomiting, which had been impossible this time.

A nasogastric tube was placed, checking its position by ultrasound. 6 liters of gastric contents were extracted within the next 48 hours. At days 2 and 4 a bedside ultrasound was performed, demonstrating the progressive decrease of gastric size. The patient was discharged after 6 days.

## Discussion

The gastric dilatation in patients with disorders of eating behavior presents a rare clinical manifestation, which may lead to gastric necrosis and perforation if not diagnosed early.

In this case, the history and physical examination did not allow suspect this pathology. Laboratory tests and conventional radiology proved insufficient.

Therefore, in case of abdominal pain of unclear etiology, we recommend an ultrasound at the bedside.

## Informed consent

The study was conducted in accordance with the ethical standards dictated by applicable law. Informed consent was obtained from each owner to enrolment in the study and to the inclusion in this article of information that could potentially lead to their identification.

